# Dietary fiber – a scoping review for Nordic Nutrition Recommendations 2023

**DOI:** 10.29219/fnr.v67.9979

**Published:** 2023-10-18

**Authors:** Harald Carlsen, Anne-Maria Pajari

**Affiliations:** 1 Faculty of Chemistry, Biotechnology and Food Science, Norwegian University of Life Sciences, Ås, Norway; 2 Department of Food and Nutrition, University of Helsinki, Helsinki, Finland

**Keywords:** dietary fiber, carbohydrates, non-communicable diseases, recommendations, polysaccharides, resistant starch, non-starch oligosaccharides, lignin, gut microbiome

## Abstract

Dietary fiber is a term crudely defined as carbohydrates (CHOs) that escape digestion and uptake in the small intestine. Lignin, which is not a CHO, is also a part of the dietary fiber definition. Dietary fibers come in different sizes and forms, with a variety of combinations of monomeric units. Health authorities worldwide have for many years recommended a diet rich in dietary fibers based on consistent findings that dietary fibers are associated with reduced incidences of major non-communicable diseases, including obesity, type 2 diabetes, cardiovascular disease, and colorectal cancer. Most fibers come from common edible foods from the plant kingdom, but fibers are also found in food additives, supplements, and breast milk. The recommended intake in Nordic Nutrition Recommendations 2012 (NNR2012) is 25 g/d for women and 35 g/d for men, whereas the actual intake is significantly lower, ranging from 16 g/d to 22 g/d in women and 18 g/d to 26 g/d in men. New studies since NNR2012 confirm the current view that dietary fiber is beneficial for health, advocating intakes of at least 25 g/day.

## Popular scientific summary

- Dietary fiber is defined as carbohydrates that escape digestion and uptake in the small intestine.
- Current intakes in the Nordic and Baltic countries vary from 16 to 22 g/d in women and 18 to 26 g/d in men.
- The main sources of fiber are whole-grain foods, fruits and berries, vegetables, nuts/seeds, and pulses.
- Evidence shows that the intake of dietary fiber is associated with lower risk of non-communicable diseases, such as cardiovascular disease (CVD) and colorectal cancer (CRC).

The term dietary fiber was first mentioned in 1953 in relation to eclampsia (1). However, building on work from three physicians (Cleave, Campbell, and Trowell) and experiences from Africa, Denis Burkitt was the first to formulate the ‘dietary fiber hypothesis’, stating that diets low in fiber increase risk of a range of diseases, including coronary heart disease, diabetes, and cancer (2). Large efforts have since been undertaken to test this hypothesis alongside defining dietary fiber.

One of the original definitions from 1972 defined dietary fibers as ‘that portion of food which is derived from cellular walls of plants which are very poorly digested by human beings’ (3). Since polysaccharides were frequently added to foods, a redefinition was proposed in 1976, by adding ‘polysaccharides and lignin’, a definition that stood for more than 30 years (4). Finally, in 2009, the latest definition from CODEX Alimentarius was proposed and largely adopted with minor modifications in most countries with the following definition (5):

*Dietary fiber means carbohydrate (CHO) polymers with 10 or more monomeric units,^1^ which are not hydrolyzed by the endogenous enzymes in the small intestine of humans and belong to the following categories:*

- *Edible CHO polymers naturally occurring in the food as consumed*
- *CHO polymers obtained from food raw material by physical, enzymatic, or chemical means^2^*
- *Synthetic CHO polymers^3^*

The European Food Safety Authority (EFSA) also includes lignin (branched aromatic alcohol), resistant oligosaccharides (3–9 monomeric units), and resistant starch in its definition. Chemical analyses of dietary fibers adhere to protocols from the Association of Official Analytical Collaboration (AOAC), and the latest protocol is AOAC 2017.16 (6).

**Fig. 1:**
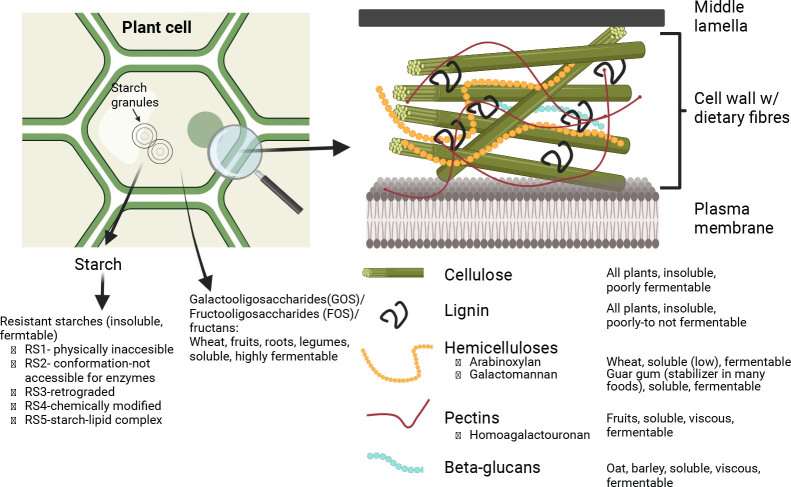
Overview of dietary fibers and their locations in the plant cell. The majority of dietary fibers are found in the cell wall where they are pivotal for structure and support. Both insoluble (cellulose and lignin) and soluble fibers are found here (e.g., hemicelluloses, β-glucans, and pectins). In the cytoplasm, starch granules are the major energy source for plant cells, and upon modification, they can become resistant starch. Also, in the cytoplasm, there are small oligosaccharides, including galactooligosaccharides (GOS), fructooligosaccharides (FOS), and fructans. The distribution of the depicted dietary fibers depends on the plant. Figure inspired by (7) and created with BioRender.com.

The majority of dietary fibers derive from cell walls of plants and are mainly structural support for the cell. Main food sources are whole grains, fruits and berries, vegetables, nut/seeds, and legumes. Additionally, several food products contain additives to improve functional properties of foods, such as thickeners and emulsion stabilization. Many of these additives are potential fibers, including guar gum, xanthan gum, alginates, carrageenans, and methylcellulose (7). As an average, whole-grain foods contain up to 12% dietary fibers (by weight), and the intake of fibers correlates strongly with cereal intake (8). Main natural dietary fibers are cellulose, hemicellulose (fibers associated with cellulose, e.g., arabinoxylans), pectins, β-glucans, and lignin. Other parts of the plant or grains contain oligosaccharides, including galactoolisaccharides (GOS; raffinose, stachyose, and verbacose from legumes), fructooligosaccharides (FOS)/fructans (e.g., inulin), or starch that may be inaccessible for digestion enzymes after ingestion because of either the food matrix preventing the access of enzymes or structural modifications of starch during processing of starch-rich foods (see Table 1 and Fig. 1). Dietary fibers are extremely varied with ~10 different monomeric CHOs used as building blocks in various combinations. The quantitatively most important building block is glucose, which is the sole monosaccharide in cellulose, resistant starch, and β-glucans. In addition, dietary fibers contain monosaccharides in various combinations, such as galactose, fructose, mannose, xylose, ribose, rhamnose, arabinose, and galactouronic acid, the latter being main building block of pectins. The structure can be straight or branched and can cross-link with other dietary fibers, including lignin. Additionally, various degrees of methylation, acetylation, and sulfation add to the complexity. Hence, a number of qualities such as size, types of bonds, monosaccharide composition, extent of lignification, and other chemical modifications will determine the physicochemical properties of the fiber, and thus the physiological effects on the host. Whereas cellulose and lignin are water insoluble and poorly fermented by colonic bacteria, hemicelluloses (to some extent), pectins, β-glucans, and non-starch oligosaccharides are water soluble and to varying degrees highly fermentable in the colon, resulting in the production of short chain fatty acids (SCFAs). Yet, other dietary fibers attract water and form a ‘gel-like’ matrices, termed viscous fibers (e.g., β-glucan). Although resistant starches are not water soluble, they are readily fermented.

**Table 1:** Overview of dietary fibers and sources

Fiber type	Sources	Main monomer(s)	Physiochemical characteristics
*Polysaccharides (DP>10)*			
*Cellulose*	Cell wall, all plants	β-Glucose (β1-6)	Insoluble, low/non-fermentable
*Arabinoxylan*	Cell wall, wheat	Xylose, arabinose	Soluble, fermentable
*Galactomannans*	Cell wall, Guar gum (food additive)	Mannose, galactose	Soluble, viscous, fermentable
*β-Glucans*	Cell wall, oat, barley	β-Glucose (β1-4)	Soluble, viscous, fermentable
*Pectins*	Cell wall, fruits, vegetables, legumes	Galactouronic acid ± various (rhamnose, xylose, galactose)	Soluble, viscous, fermentable
*Resistant starches*			
*RS1-physically inaccessible*	Whole grains	α-Glucose (α1-4)	Insoluble, fermentable
*RS2-conformation*	Raw vegetables, cereals	α-Glucose (α1-4)	Low solubility, fermentable
*RS3-retrograded after cooking and cooling*	Any starch source	α-Glucose (α1-4)	Low solubility, fermentable
*RS4-chemically modified*	Any starch-e.g., acetylated	α-Glucose (α1-4)	Low solubility, fermentable
*RS5-starch-lipid complex*	Any starch source-e.g., amylose-stearic acid complex	α-Glucose (α1-4)	Medium solubility, fermentable
*Non-starch oligosaccharides (DP 3*-*9)*			
*Inulin/FOS*	Fruits, vegetables, wheat	Fructose + glucose	Soluble, fermentable
*GOS (stachyose, raffinose, verbacose)*	Legumes	Galactose+ sucrose	Soluble, fermentable
*Non-carbohydrate (large polymers)*			
*Lignin*	Cell wall, all plants	Phenols	Insoluble, low/non-fermentable

FOS, fructooligosaccharides; GOS, galactoolisaccharides.

AOAC develops analytical methods to quantify dietary fibers, with AOAC protocols regularly adjusted (3). Analysis involves *in vitro* digestion of food products with amylase and proteases and separation into water insoluble and water-soluble fibers and a further separation into high- and low-molecular weight soluble fibers combined with high-performance liquid chromatography (HPLC) methods. The latest protocol is based on AOAC 2017.16 (6, 9). Numerous experimental and observational studies have been carried out over the last two-three decades, aiming to elucidate health effects and the underlying mechanisms of dietary fibers. A general consensus is that dietary fibers are beneficial with respect to reducing risks of obesity, type 2 diabetes (T2D), cardiovascular diseases (CVD), and certain cancers (i.e., colorectal cancer [CRC] and all-cause mortality.

The aim of this scoping review is to describe the totality of evidence for the role of dietary fibers for health-related outcomes as a basis for setting and updating the dietary guidelines for fiber intake (Box 1).

Box 1 Background papers for Nordic Nutrition Recommendations 2023

- This paper is one of many scoping reviews commissioned as part of the Nordic Nutrition Recommendations 2023 (NNR2023) project (10).
- The papers are included in the extended NNR2023 report, but, for transparency, these scoping reviews are also published in Food & Nutrition Research.
- The scoping reviews have been peer reviewed by independent experts in the research field according to the standard procedures of the journal.
- The scoping reviews have also been subjected to public consultations (see report to be published by the NNR2023 project).
- The NNR2023 committee has served as the editorial board.
- While these papers are a main fundament, the NNR2023 committee has the sole responsibility for setting dietary reference values in the NNR2023 project.

## Methods

This review follows the protocol developed within the NNR2023 project (Box 1) (10). The sources of evidence used in this scoping review follow the eligibility criteria described in the paper ‘The Nordic Nutrition Recommendations 2022 – Principles and methodologies’, published in *Food & Nutrition Research* (2020) (11). Literature searches were conducted with the aim of covering relevant literature from 2012 to date. Search string was (‘fiber’ [Title] OR ‘fibre’ [Title] AND diet*) AND (‘2011’ [Date – Publication] : ‘3000’ [Date – Publication]) AND Humans [Filter] AND Review [Publication Type]. General literature search in PubMed on 24th of September 2019 resulted in 157 hits/reviews, which were limited to 40 (based on titles or abstracts), which formed the basis for this review. A new search was performed in October 2022, and the very latest literature search was done on March 1, 2023. Based on screening of the abstracts, no new literature that needed to be included was found. The articles forming the basis of the present review of health effects are listed in Table 2.

**Table 2:** Overview of studies used to assess health effects relevant for the Nordic and Baltic countries

Outcome and studies	Study type	Main findings/conclusions
**Body weight/appetite**		
**Wanders et al. (68)**	Systematic review, clinical trials	Short-term appetite, energy intake and body weight were reduced by viscous fibers. Long-term effects were not dependent on specific fibers indicating multiple mechanisms conferred by fibers. Weight reduction was moderate (~0.4 kg/4 wk intervention).
**Cho et al. (69)**	Systematic review, prospective studies	High vs low cereal fiber intake over 6.5–8 years either reduced body weight gain or reduced weight. Modest effects in absolute numbers.
**Reynolds et al. (70)**	Meta-analysis, clinical trials	Body weight reduced moderately, when comparing high vs low fiber intake long term (duration of intervention variable).
**Type 2 diabetes**		
**Reynolds et al. (70)**	Meta-analysis, prospective studies	Incidence of T2D was significantly reduced (16%) by high vs low fiber intake.
**Veronese et al. (71)**	Umbrella review, prospective studies	Incidence of T2D was significantly reduced (17%) by high vs low fiber intake. Evidence judged as suggestive.
**Davison et al. (72)**	Meta-analysis, prospective and clinical trials	Cereal fibers but not fibers from fruit and vegetables significantly reduced incidence of T2D.
**Cardiovascular disease (CVD), stroke, and coronary artery disease (CAD)**		
**Reynolds et al. (70)**	Meta-analysis, prospective cohorts, CVD, CAD, stroke	Dose-dependent decrease in CVD and CAD incidence and mortality. Stroke incidence reduced but evidence weak compared to CVD and CAD.
**Veronese et al. (71)**	Umbrella review, prospective studies	CVD mortality, CVD incidence, and CAD incidence significantly reduced by high- vs low-fiber intake. Evidence judged to be strong. For stroke, fiber reduces incidence, but evidence judged to be weak.
**Threapleton et al. (73)**	Systematic review and meta-analysis, of prospective studies	Total dietary fiber dose-dependently reduces incidence of first stroke and judged to be convincing despite high degree of heterogeneity in studies.
***Hypertension***		
**Evans et al. (74)**	Systematic review and meta-analysis of clinical trials	Non-significant decrease in blood pressure when all fibers were studied combined. Stronger and significant effects of β-glucans (SBP -2.7, DBP -1.45 mmHg)
**Reynolds et al. (70)**	Meta-analysis, clinical trials	Dose-dependent decrease in systolic blood pressure with total dietary fiber (-1.27 mmHg)
***Cholesterol***		
**Reynolds et al. (70)**	Meta-analysis, clinical trials	Highest fiber reduced total cholesterol by 0.15 mmol/L (95% CI -0.22 to -0.07).
**Cancers**		
**WCRF/AACR (75)**	Meta-analysis, prospective cohorts	High vs low total dietary fiber. 9% reduced risk of CRC, but no evidence for a reduction in other cancers.
**Reynolds et al. (70)**	Meta-analysis, prospective cohorts	Highest vs lowest intakes and overall cancer mortality and CRC were significantly reduced by 13% and 16%, respectively. Modest reduction on breast cancer (7%). No effect on other cancers.
**Inflammatory bowel disease (IBD)**		
**Liu et al. (76)**	Meta-analysis, prospective cohorts	Significant reduction of developing Crohn’s disease (66% on overage). No or inconclusive effect on ulcerative colitis. Evidence for Crohn’s disease judged to be weak.
**All-cause mortality**		
**Reynolds et al. (70)**	Meta-analysis of prospective studies	High vs low total dietary fiber reduces age-adjusted all-cause mortality by 15%. Dose-dependent decrease in mortality.
**Veronese et al. (71)**	Umbrella review, prospective studies	High vs low total dietary fiber reduces age-adjusted all-cause mortality by 16%.
**Hajishafiee et al. (77)**	Systematic review and meta-analysis, prospective studies	High vs low intake of cereal fiber reduced all-cause mortality by 19%. Dose-dependent decrease in all-cause mortality by cereal fiber.
**Kim et al. (78)**	Meta-analysis of prospective studies	High vs low total dietary fiber reduced all-cause mortality by 23%.

WCRF/AICR, World Cancer Research Fund/American Association for Cancer Research.

*A de novo* Scoping review (SR) on dietary fiber and growth, iron status, and bowel function in children 0–5 years old, commissioned by NNR, was integrated and discussed in the review.

## Physiology

Although dietary fiber per definition passes through the small intestine (SI) to the colon, dietary fibers affect both upper and lower gastrointestinal tract (GI) tract (7). Most fiber-rich foods contain soluble, insoluble, viscous, and fermentable fibers and are therefore part of the normal food matrix entering the GI tract with different properties related to function. Food entering the mouth is within minutes transported to the stomach, where soluble and viscous fibers attract water and cause swelling and delayed gastric emptying that can affect satiety and nutrient uptake (12). In the SI, swelling, viscosity, and so-called bulking can lead to increased transit time through a mechanism coined ileal brake (13) and optimize nutrient uptake. Viscosity, caused primarily by soluble fibers such as β-glucans from oats and barley, can also lead to reduced nutrient uptake, with a resultant reduced postprandial glucose rise (14) and lipids (15–17). It has also been demonstrated that dietary fibers can reduce digestibility of fats and proteins (18, 19). Also, 95% of the bile acids are usually reabsorbed in the distal part of the SI, which is reduced by viscous fibers (e.g., β-glucans). Since bile acids derive from cholesterol, reduced uptake of these molecules by β-glucans is now accepted as the main mechanism by fibers for reducing cholesterol in blood (20).

### Effect on mineral uptake, transit time, and energy contribution

Uptake of iron and calcium is also affected by dietary fibers and/or fiber-rich foods. Cereal bran, for instance, contains phytate, which is known to sequester iron, zinc, and calcium and discourage uptake of these minerals (21). However, increased uptake of calcium in the colon can be facilitated by oligosaccharides, including fructans (22, 23) and galacto-oligosaccharides (24), possibly due to a reduced pH caused by SCFA production promoted by these fibers.

Some concerns have been raised that the interference of fiber in the absorption of iron and other key nutrients in the SI may compromise adequate energy and micronutrient uptakes in children, leading to problems in growth and development (25). The systematic review commissioned by the NNR2023 committee addressed this issue and found no clear association between high intake of dietary fiber and growth among children of 0–5 years of age, while the lack of studies leaves the issue of fiber intake on iron status unsolved (26). Two earlier systematic reviews (27, 28) covering ages from early years to adulthood found no indication for adverse effects of high-fiber diets in affluent countries either.

Insoluble and low- to non-fermentable fibers such as cellulose and lignin are important for bulking, i.e., they increase the volume of the GI content and thereby affect transit time from ingestion to evacuation as faces, as shown after the intake of wheat bran (29, 30). Insoluble and low- to non-fermentable fibers are responsible for this effect as opposed to readily fermentable fibers, which do not contribute to bulking to same degree as they are not intact throughout the colon passage (31). The relevance is that reduced transit time prevents constipation in healthy individuals (31). For other gastrointestinal disorders with constipation as part of the symptoms (e.g., IBS), national and international guidelines provide some recommendations for the management and treatment but are not specific regarding doses or types of fibers (32). For preventing cancer, faster transit rate can shorten the time for potential carcinogens to act on colonocytes, which can develop into adenomas (33).

The net energy contribution from dietary fibers to the host is on average 8 kJ/g fiber, but this number depends on the nature of the dietary fiber, especially with respect to fermentability. Also reduced uptake of energy-yielding nutrients will affect how much energy is provided by dietary fibers. Hence, 8 kJ/g energy assumes an intake of a variety of dietary fibers, including both fermentable and non-fermentable types. For instance, the intake of the easily fermentable oligosaccharide inulin can provide ~12 kJ/g (34), whereas non-fermentable dietary fibers with ‘anti-nutrient’ properties, in fact, can contribute negatively to the energy balance (19, 35, 36). Intakes of 20–25 g/day can thus on average provide ~2–3% of the total energy intake (37).

### Fermentation and SCFAs

**Fig. 2:**
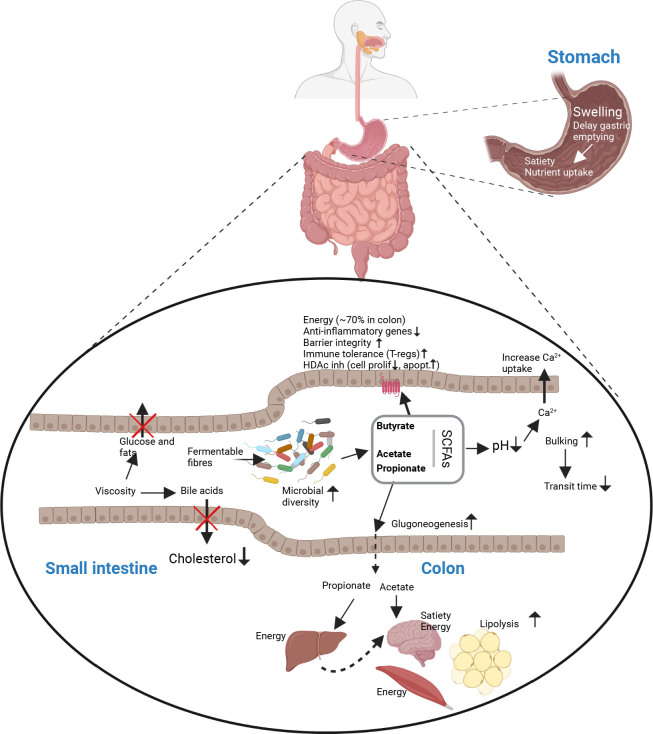
Mechanisms of dietary fibers in the gastrointestinal tract. In the stomach and SI, swelling and viscosity of fibers affect transit time, nutrient uptake, and bile acid reuptake. Colonic short chain fatty acids (acetate, propionate and butyrate) are made through fiber-fermentation by commensal bacteria. Butyrate provides energy for colon epithelial cells and affects cell signaling and immune tolerance, whereas acetate and propionate are taken up into the blood to exert various functions including energy provisions and possibly satiety in the brain satiety center. Figure inspired by (7) and created with BioRender.com.

Although all dietary fibers including cellulose and lignin (low fermentability) can be fermented by colonic bacteria, the most fermentable dietary fibers are the short soluble oligosaccharides, including inulin, FOS and GOS, and larger soluble fibers, such as pectin, β-glucans, arabinoxylans, and galactomannans. Also, resistant starches are readily fermented despite their low solubility (7). Fermentable dietary fibers are metabolized by anaerobic colonic bacteria, which can produce substantial amounts of SCFAs, most prominently acetate, propionate, and butyrate. On average, around 20 g of SCFAs are produced daily in humans (38, 39), with 90–95% being absorbed in the colon (40). Highest concentrations are found in the proximal part of the colon, and concentrations of acetate, propionate, and butyrate can reach 60, 20, and 20 mmol/kg colonic content, respectively (41). The amount produced of respective SCFAs, especially propionate and butyrate, is affected by the fiber source (42). Butyrate primarily acts locally and provides ~70% of energy for colonic epithelial cells (43). Butyrate can also bind to at least 3 different G-protein coupled receptors (GPCRs): GPR43/*FFAR2*, GPR41/*FFAR3*, and GPR109. In all cases, binding to these receptors elicits various intracellular responses that can affect cellular proliferation and apoptosis, reduce inflammation, improve cellular repair, and strengthen barrier functions in the colonic epithelium (44). Furthermore, butyrate can act as a histone deacetylase inhibitor (HDACi) in colon cancer cells and regulate genes involved in cell proliferation (reduced) and apoptosis (increased) (33). For these reasons, butyrate is highlighted as key in the protection against CRC and gut inflammation, which have been demonstrated in many animal studies (45). Additionally, butyrate is important for immune tolerance toward food antigens and commensal bacteria. Butyrate can activate FOXP3, a transcriptional regulator specific to regulatory T cells, and stimulate their differentiation residing in the immune compartment of the gut mucosa (Lamina propria) (46). Acetate, especially, and propionate are more readily distributed systemically after uptake from the colon and are used as energy in the brain and muscle (acetate) or in the liver (propionate) (47). However, both acetate and propionate can have regulatory roles, particularly in relation to energy homeostasis and appetite regulation. Acetate can directly suppress appetite through central hypothalamic mechanisms with a resultant reduction in food intake (48). Both butyrate and propionate can stimulate intestinal gluconeogenesis (IGN) directly in the colonocytes (butyrate) or on gut associated neurons (propionate) and also stimulate IGN via CNS-regulated mechanisms (45, 49–51). Observed consequences of IGN in animal models are reduced food intake (satiation) and alleviation of fatty liver in high-fat fed mice (52) (see also Fig. 2).

In addition to producing SCFAs with a plethora of biological effects, dietary fibers affect the composition of gut microbiota. Especially, greater diversity of bacterial species has been associated with improved health linked to, e.g., resilience against pathogenic bacteria, improved intestinal barrier integrity, and metabolic health (53). In mice, a fiber-deprived diet can, e.g., lead to higher abundance of bacteria using mucus as food source, resulting in thinner mucus and higher vulnerability to pathogenic infections (54).

Most of the mechanisms and health-beneficial effects of SCFAs have been demonstrated in animal models, and human studies are limited. However, in one human study, the supplementation of propionate to overweight and obese subjects for ~6 months results in a significant reduction in body weight gain compared to controls (55). Studies investigating glucose homeostasis also show overall beneficial effects on fasting glucose levels and insulin response (56, 57), whereas other studies have yielded no effect on the same parameters (58). Although studies exist that have investigated health effects by SCFA, a European expert group concluded that despite the overwhelming mechanistic evidence of beneficial effects on gut and metabolic health conferred by SCFAs derived from fiber fermentation, future studies in humans are warranted (45).

## Dietary fibers and biomarkers of intake

As SCFAs are considered the most abundant metabolites of gut fermentation of dietary fiber, one would assume them to present a good biomarker for dietary fiber intake. This, however, is not the case even though SCFA can be measured in the stool and blood (41). The main reason for this is that SCFAs are rapidly and almost completely absorbed and metabolized either in the colon by the colonocytes (butyrate) or in the liver and other organs (propionate and acetate) (45). Thus, the concentration measured in the faces represents the remaining 5–10% of the total SCFA produced in the colon. Similarly, plasma SCFA concentrations reflect the net result of production, absorption, and utilization by the host, not directly the colonic fermentation. Nevertheless, faecal concentrations have frequently been used as a measure of SCFA production by fiber in human intervention studies (59), and plasma SCFAs have also been shown to be affected by dietary fibers in mechanistical studies (60, 61). In general, plasma concentrations of SCFA are substantially lower than in the colonic lumen (62–65). A further complication using SCFA as a biomarker is related to the fact that SCFAs are highly volatile compounds and easily lost during sample storage (66). A verification between dietary fiber intake and SCFA either in the plasma or faeces in population studies is still lacking. Other more indirect biomarkers for fiber intake can be considered. Plasma and urine alkylresorcinols (ARs) and their metabolites are fairly well-established biomarkers for the consumption of whole-grain rye and wheat, as reviewed by Landberg and coworkers (67). ARs are also associated with cereal fiber intake in several studies, indicating that they could be useful indicators of fiber intake particularly in Nordic countries, where whole grains are the major fiber source.

## Dietary intake in Nordic and Baltic countries

Daily mean intakes of dietary fiber vary within the range of 16–26 g/d among adults including both sexes, the highest intake of 26 g/d being observed among Norwegian men (Table 3a and b). Intakes are below the recommended minimum level of 25 g–35 g/d in all Nordic and Baltic countries. Among children, the intakes vary in the range of 13–21 g/d, even though the data are lacking altogether in Latvia and are not covering all age groups in other countries (Table 4a and b). Identifying and comparing the major dietary fiber sources among the Nordic and Baltic countries is somewhat challenging as food grouping differs between the countries. In Denmark, Finland, and Norway, bread and cereals account for around 50% of fiber sources in women and men, while in Sweden, their contribution is little less (slightly over 30%). Vegetables, fruit, and berries and to a lesser extent potatoes and potato dishes cover the rest of fiber sources in all the mentioned countries.

**Table 3:** Daily mean intakes of fiber among adults in Nordic and Baltic countries (79)

Table 3 A															
		Denmark 2011 (18–75 years)			Finland 2017 (18–74 years)			Iceland 2010 (18–80 years)		Norway 2010 (18–70 years)			Sweden 2010 (18–80 years)		
		Men (*n* = 1,464)	Women (*n* = 1,552)		Men (*n* = 780)	Women (*n* = 875)		Men (*n* = 632)	Women (*n* = 680)	Men (*n* = 862)		Women (*n* = 925)	Men (*n* = 792)		Women (*n* = 1,005)
**Fiber g/d**		24	21		22	20		18	16	26		22	21		19
**Fiber g/MJ**		2.2	2.5		2.5	2.9		1.8	2.2	2.5		2.9	2.3		2.6
**Table 3 B**															
	**Estonia 2014 (18–74 years)**						**Latvia 2018 (19–64 years)**				**Lithuania 2019 (19–75 years)**				
	**Men**			**Women**			**Men**		**Women**		**Men**			**Women**	
**Fiber g/d**	19			17			20		18		18			16	
**Fiber g/MJ**	2.2			2.6			–		–		1.9			2.2	

**Table 4a:** Daily mean intakes of fiber (g/MJ) among children in Nordic and Baltic countries (79)

Denmark			Finland			Norway		Sweden		
*Age, y*	Boys	Girls	*Age, y*	Boys	Girls	*Age, y*	Both boys and girls	*Age, y*	Boys	Girls
*4–5*	2.6	2.6	*3–4*	2.4	2.4					
*6–9*	2.4	2.5	*5–6*	2.4	2.4					
*10–13*	2.1	2.2				*10–11*	16*	*12*	2.0	2.1
*14–17*	2.0	2.1				*14–15*	16*	*15*	2.0	2.2

* Intake given as g/d as the data in g/MJ are not available.

**Table 4b:** Daily mean intakes of fiber (g/MJ) among children in Nordic and Baltic countries (79)

Estonia			Lithuania	
*Age, y*	Boys	Girls	*Age, y*	Both boys and girls
*2–5*	2.3	2.3		
*6–9*	2.1	2.2	*7–10*	2.0
*10–13*	2.0	2.1	*11–14*	1.9
*14–17*	2.2	2.2	*15–18 (19)*	1.7

## Health outcomes relevant for Nordic and Baltic countries

### Body weight/obesity and appetite

Two systematic reviews (68, 69) and one meta-analysis (70) were included in this summary. The first systematic review by Wanders et al. (68) examined 104 controlled clinical trials focusing on subjective appetite (short term), energy intake (short- or long term), and/or body weight (long term). A major interest was to investigate effects conferred by fibers with different physiochemical properties (viscosity, solubility, and fermentability). Subjective appetite scored by a visual analogue scale (VAS) 1–4 h after ingestion of selected fibers revealed an overall fiber-mediated reduction in appetite. From 43 identified studies (58 comparisons), 43% of the comparisons (fiber vs control) reported a relevant reduced subjective appetite (>10% reduction). Mean appetite score was reduced by 5% in the studies combined. Viscous fibers (pectins, mannan, and glucans) gave the greatest reduction (~17% reduced appetite). In line with these results, short-term energy intake was overall reduced by dietary fibers, especially viscous fibers, as measured by recording the intake of an *ad libitum* test meal within hours after ingestion of a defined dietary fiber. Viscous fibers compared to non-viscous fibers gave the strongest reduction in energy intake (0.1–0.4 MJ reduction compared to control). Long-term studies were separated into energy intake and body weight changes during defined fiber interventions (study durations from 3 to 19 weeks, average 8.4 and 11.1 weeks, respectively). Food and energy intakes were assessed by different methods, including 24h recall, 7 d food history, food frequency questionnaires, and, in some cases, weighed food records. Both energy intake and body weight were lowered by dietary fibers, but in contrast to the short-term studies, the effect was not confined to any specific fiber type, indicating that long-term effects of dietary fibers are more diverse, involving multiple mechanisms, including fermentation with putative long-term effects of bacterially derived SCFAs. The mean lower energy intake per day was 0.15 MJ (2.6% of daily energy intake) compared to control. Body weight was compared between different time points and baseline, with a mean body weight reduction of 0.39 kg/4 week of intervention. The latter findings agree with a more recent comprehensive meta-analysis authored by Reynolds and coworkers and commissioned by World Health Organization (WHO) (70), in which 27 controlled trials concluded with a weight reduction of 0.37 kg as a result of increased fiber intake. Importantly, the studies reporting on long-term effects on body weight were judged to be of high quality with a large number of studies and uniform methodology.

In the second systematic review by Cho and coworkers (69), the aim was to examine the effect of cereal fibers on body weight by comparing body weight development over a defined period of time. Two prospective cohort studies were analyzed: the Health Professionals’ Follow-Up study (HPFS, 27,082 participants) (80) and The European Diogenes project (89,432 participants) (81), with subjects followed 8 and 6.5 years, respectively. Both studies demonstrate significant, but modest effects on body weight by cereal fibers. In the HPFS, body weight in those with the highest cereal fiber intake increased less than those ingesting the lowest amounts over the 8 years period (0.91 kg increase vs 1.3 kg increase; 0.39-kg difference). In the Diogenes project, body weights were on average 77 g/year lower for each 10 g increase in fiber intake. In sum, both these prospective cohort studies demonstrate a modest, although significant weight/weight gain reduction by higher intakes of dietary fibers. The authors conclude that the level of evidence for weight reduction with higher intake of fibers is moderate to limited for cereal fibers and mixtures of whole grains and bran and limited to inadequate for whole grains.

Conclusion: Dietary fibers lower subjective appetite scores, acute energy intake, long-term energy intake, and body weight. Short-term studies indicate a more pronounced effect by viscous fibers compared to fibers with other physiochemical properties. Long-term effects are not confined to specific fibers, suggesting that multiple mechanisms are responsible for reduced body weight and energy intake. Effects on body weight by increasing fiber intakes are significant and robust but modest in absolute numbers.

### Type 2 diabetes

A comprehensive report by Reynolds and coworkers, published in *The Lancet* in 2019 (70), compared both the highest vs lowest fiber intakes and dose–response relationship with respect to T2D incidence in 17 prospective studies. Highest vs lowest intake reduced relative risk (RR) significantly by 16% (RR 0.84, 95% CI 0.78–0.9), and a dose–response analysis demonstrated that 20–24 g/d provided the best benefit. Cereal fiber intake largely mirrored the conclusions for total dietary fiber intake (70). These findings are also consistent with another large umbrella analysis by Veronese and coworkers (71) and a systematic review by Davison et al. (72). The Veronese report from 2018 reviewed prospective studies and concluded that the incidence of T2D is inversely associated with the high-fiber intake vs low intake (RR 0.882, 95% CI 0.73–0.93), but categorized their findings as only suggestive for a protective effect. The last study was as systematic review that scrutinized whether different types of fibers differentially affected incidence of T2Ds, concluding that the evidence for beneficial effects of cereal fibers was strong, whereas the evidence for fibers from vegetables and fruits was weak and less consistent (72).

Conclusion: Dietary fibers reduce the incidence of developing T2D, although some studies judged the evidence as weak. Evidence for a protective effect may be stronger for the intake of cereal fibers and inconsistent with respect to fibers from vegetables and fruits.

### CVDs including hypertension and cholesterol

Three large reviews have been included with respect to risk assessments of CVD, coronary artery disease (CAD), and stroke: one umbrella review from 2018 scrutinizes 18 meta-analyses (from 298 prospective studies) (71). The other is a meta-analysis (WHO report) by Reynolds et al. (70), and the third study is a systematic review and meta-analysis specifically on stroke (73). Outcomes in the two first studies were CVD, CVD mortality, stroke, and CAD. RRs for the highest fiber intake vs lowest were calculated. The umbrella review concludes that highest intake of dietary fibers significantly reduces CVD incidence by 9% (RR 0.91; 95% CI 0.89, 0.93), CVD mortality by 19% (RR 0.81; 95% CI 0.78, 0.86), stroke by 17% (RR 0.83; 95% CI 0.74, 0.93), and CAD by 7% (RR 0.93 95% CI 0.9, 0.96). The strongest level of evidence was found for CVD mortality, CVD incidence, and CAD, whereas for stroke, the level of evidence was found to be weak compared to CAD (71). The WHO report corroborates the conclusions from the aforementioned umbrella review. In the WHO study, they also included analyses of dose–response for CAD and found a significant inverse dose–response relationship between fiber intake and risk of CAD (70). Threapleton et al. (73) investigated the association of dietary fibers specifically to stroke, and in line with the studies discussed earlier, they find a dose-dependent reduction in risk of stroke (7% when assessing incremental intakes of 7 g/day, RR 0.93; 95% CI 0.88–0.98). A weakness in many of the studies on stroke is high degree of heterogeneity. Therefore, firm conclusions regarding the intake of dietary fiber and risk of stroke warrant more studies.

### Hypertension

Two systematic reviews investigated dietary fiber intakes in clinical trials (70, 74). The first, published in 2015 (74), included 28 studies investigating the effects of different fiber types on systolic and diastolic blood pressure (BP). Overall effects of total dietary fiber revealed a non-significant reduction in BP (systolic BP, -0.92 mmHg, 95% CI -2.28–0.63 mmHg, diastolic BP -0.71, 95% CI -1.90–0.47). However, β-glucan/β-glucan rich foods significantly reduced both systolic (-2.7 mmHg) and diastolic BP (-1.45 mmHg) (74). In the WHO report by Reynolds et al., 15 clinical trials were investigated, in which different ranges of high-fiber intakes (0–25 g/d, 25–30 g/d, 30–35 g/d, and >35 g/d) were analyzed. High versus low intakes were modeled to estimate RR in these two scenarios (high vs low). Their conclusion was that high vs low intake of dietary fibers, regardless of fiber type, significantly reduced systolic BP (on average -1.3 mmHg, 95% CI -2.5 to -0.04 mmHg) (70).

### Cholesterol

One meta-analysis study was included the already mentioned Reynolds publication (70). In their study, 36 clinical trials were identified, which compared blood cholesterol between those with highest to those with lowest dietary fiber intake following interventions. In the highest fiber intake groups, the reduction in total cholesterol was 0.15 mmol/L (95% CI -0.22 to -0.07).

Conclusion: New studies confirm previous observations that high intake of dietary fibers is protective against CVD, which comprises CAD and stroke. Whereas the evidence is strong for CAD, the evidence for stroke is judged to be less conclusive. When taking clinical trials assessing hypertension and cholesterol into account, the overall conclusion is that high versus low intake of dietary fibers is associated with reduced risk of CVD.

### Cancers

Numerous studies have investigated the association between a high-fiber intake and reduction in CRC risk. In their latest report from the Continuous Update Panel (CUP) in 2018 (75), World Cancer Research Fund/ American Association for Cancer Research (WCRF/AACR) summarized this topic by including 23 studies, in which 21 studies were used for a meta-analysis with 16,562 cases. They maintain their conclusion from previous reports that there is strong evidence supporting that foods with dietary fibers *probably protect against developing CRC*. Dose–response relationship was also evaluated. Although large heterogeneity was observed among the studies, the RR of CRC incidence was reduced in a dose-dependent manner (RR 0.91; 95% CI 0.88, 0.94). For other cancers including breast, endometrial, gastric, and esophageal cancer, limited or no evidence of a reduction was noted.

The WCFR/AACR conclusion is backed up by the recent and comprehensive systematic review and meta-analyses from Reynolds et al. (70), where 185 prospective studies and 58 clinical trials were included. Outcomes were overall cancer mortality and incidences of CRC, breast cancer, prostate cancer, and endometrial cancer. When comparing highest to lowest intakes, overall cancer mortality and CRC were significantly reduced by 13% (RR 0.87; 95% CI 0.79, 0.95) and 16% (RR 0.84; 95% CI 0.78, 0.89), respectively. A clear dose-response relationship was also evident for cancer mortality and CRC incidence. For other cancers, a moderate decreased risk in breast cancer incidence was observed (RR 0.93; 95% CI 0.90, 0.97). For prostate and endometrial cancer, no inverse relationship was noted for increased fiber intake.

Conclusion: New studies confirm earlier findings that the association between reduction in overall cancer mortality and CRC incidence and dietary fibers is convincing. Evidence for breast cancers is limited suggestive. For other cancers, no relationship with fiber intake was found.

### Inflammatory bowel disease

One meta-analysis has reviewed intake of dietary fibers and the risk of developing inflammatory bowel disease (IBD) (ulcerative colitis and Crohn’s disease) based on eight observational studies (one cohort study, six case–control studies, and one nested case–control study) (76). For Crohn’s disease, seven studies were identified (1 cohort and 6 case–control). Three of the seven studies demonstrated significantly reduced risk of Crohn’s disease, whereas the remaining four showed no significant effect when highest versus lowest fiber intakes were compared. The overall effect showed RR 0.44 (95% CI 0.24, 0.68). For ulcerative colitis, although borderline, no significant effect of dietary fibers was observed (RR 0.80; 95% CI 0.64, 1.00).

Conclusion: Dietary fibers may reduce the risk of developing IBD, especially Crohn’s disease. Due to inconsistency among the different studies, the evidence is judged to be weak for Crohn’s disease and not conclusive for ulcerative colitis.

### All-cause mortality

Several extensive meta-analyses and systematic reviews have investigated the association between fiber intake and all-cause mortality (70, 71, 77, 78). In all these reports, the highest quintile of dietary fiber intake was compared with the lowest quintile of intake. On average, the different reviews report a reduced RR of 15–20% for all-cause mortality. The most comprehensive report, commissioned by WHO, included about 135 million person-years of data from 185 prospective studies and 58 clinical trials. They observed a 15% RR reduction for all-cause mortality (80,139 cases included) of higher compared with lower intakes of total dietary fiber (RR 0.85, 95% CI 0.79, 0.91). The WHO report also found a significant dose-dependent decrease (70).

Conclusion: There is convincing evidence that higher vs lower intake of dietary fiber is associated with reduced risk of age-adjusted all-cause mortality (71).

## Requirement and recommended intakes

A huge body of evidence over many years consistently report on health-beneficial effects of a higher intake of dietary fibers, and the conclusions from the NNR2012 are largely unchanged. The strongest evidence is related to all-cause mortality followed by coronary heart disease and CRC. Evidence for a protective effect against stroke and T2D is judged to be significant, but still weaker than for all-cause mortality and incidences of coronary heart disease and CRC. Effects on body weight are judged significant, but modest. For IBD, dietary fibers may be protective, but too few studies have investigated this relationship to draw a firm conclusion.

The consistency between the controlled trials and prospective study results, together with the dose-response relationships, provides support that the effect on cardiometabolic diseases is likely to be causal and not a consequence of confounding variables. For several of these outcomes, the dose-response is linear (70). Overall, based on observational and experimental studies, to include dietary fibers as a vital part of the dietary plan is clearly advisable. Intakes should be at least 25–29 g per day, with data indicating additional benefits with higher intakes (70). NNR2012 recommendations are 25 g/day for women and 35 g/day for men, or more precisely 3.0 g/MJ energy (12.5 g/1,000 kcal). To reach an intake of at least 25 g/day, energy intake should be 8.3 MJ/day, but in the Nordic countries, the average energy intake in women is 7.3 MJ/d (range 6.5–8). Taking these numbers into account, to reach intake levels of 25 g/d or more, the recommended intake based on energy intake should be at least 3.0 g/MJ (25 g/d in women and 35 g/d in men). These numbers are well above the actual intakes, which, in the Nordic and Baltic countries, range from 16 g/d to 22 g/d in women and 18 g/d to 26 g/d in men. It appears that fibers from cereals largely reflect the conclusions drawn from total fiber intakes, which makes sense since on average, 50% of the fiber intake comes from cereals. To what extent other dietary constituents associated with cereals play a role is not clear, but it is pertinent to speculate that phytochemicals such as polyphenols associated with dietary fibers may influence several of the outcomes (82). Data regarding specific sources (e.g., cereals, legumes, fruits, or vegetables) or subcategories (e.g., soluble, insoluble, or extracted) are more limited. Dietary intake data of fibers in most prospective studies do not discriminate between fiber types, explained mainly by the fact that most food composition databases in which food intake data are drawn have either not included fiber subcategories in their composition tables or that chemical analyses (AOAC protocols) at the time of most studies have been carried out have not been updated to the current standard (3). Future studies will likely provide more precise data on fiber type in relation to health effects.

The evidence gathered from observational and clinical studies is largely supported and explained by mechanistic studies, primarily done in animal models. Many of the dietary fibers are fermented by colonic bacteria, producing three main SCFAs with different effects on the host such as reduction of appetite, improving glucose regulation, stimulation of immune tolerance, reducing inflammation, and regulation of cell proliferation with consequences for cancer development. But dietary fibers also provide other important effects, such as bulking leading to satiety, shorter transit time, alleviation of constipation, impact on nutrient uptake, and likely a beneficial effect on CRC prevention. Viscous fibers can also affect numerous reactions including reabsorption of bile acids resulting in reduced cholesterol and can also lead to slower uptake of glucose and lipids after a meal (7). It is therefore plausible that a variation of fibers with different physiochemical properties is key for the wide range of benefits offered by dietary fibers.

A limited number of studies on fiber intake and children’s health are available, hampering definite conclusions to be made on recommended fiber intake in children. However, it seems unlikely that higher fiber intakes than are currently consumed would result in impaired growth in children on omnivorous diets in affluent societies such as Nordic and Baltic countries. On the contrary, indication of health benefits including lower risk of constipation, obesity, high blood lipids and BP, metabolic syndrome, and insulin resistance seems likely.

## Footnotes

1 Allows international authorities to decide whether those compounds with DP of 3–9 would be allowed.

2,3 For the isolated or synthetic fibers, they must show a proven physiological benefit to health as demonstrated by generally accepted scientific evidence to competent authorities.
